# Supporting people living with HIV in serodiscordant partnerships to attempt a desired pregnancy by integrating sexual and reproductive health and HIV interventions

**DOI:** 10.7448/IAS.20.2.21829

**Published:** 2017-03-08

**Authors:** Manjulaa Narasimhan, Connie Celum, Ian Askew, James Kiarie, Sheryl van der Poel

**Affiliations:** ^a^ Department of Reproductive Health and Research, World Health Organization, Geneva, Switzerland; ^b^ International Clinical Research Center, School of Public Health, University of Washington, Seattle, WA, USA; ^c^ Independent Researcher, Geneva, Switzerland; ^d^ Population Council, Washington, DC, USA

**Keywords:** HIV, fertility care, antiretroviral, PrEP, infertility, IVF/ICSI, reproductive health, reproductive rights

## Introduction

Globally, in 2015, an estimated 36.7 million people were living with HIV, and up to half have HIV-negative partners (i.e. they are in serodiscordant relationships) [1,2]. The desire to have children expressed by people living with HIV or in serodiscordant relationships has often been inadequately supported by existing HIV and sexual and reproductive health services [3,4]. For all serodiscordant couples, regardless of whether or not they have fertility problems, fertility screening and management interventions are essential in order to minimize the risk of HIV transmission while taking steps to achieve pregnancy; for serodiscordant couples who also have fertility problems, such interventions are absolutely critical. There are several key principles that should underpin the design and provision of fertility care for people in serodiscordant relationships. In delivering counselling and services, healthcare providers should support informed, voluntary decision-making about reproductive choices, to create environments that reduce stigma associated with HIV and fertility problems or infertility, as well as to encourage safe, voluntary disclosure of HIV status, fertility intentions and desires to partners [5].

The WHO-consolidated guideline on sexual and reproductive health and rights of women living with HIV includes several options to support achieving pregnancy with minimal risk of HIV transmission in HIV-serodiscordant relationships [6]. These include (a) antiretroviral therapy (ART) use by the person living with HIV to suppress viral load; (b) use of oral pre-exposure prophylaxis (PrEP) by the partner who does not have HIV, which are also interventions that are important for health and well-being beyond attempting pregnancy; (c) condom-less intercourse during the woman's peak fertile days; (d) screening and treatment of sexually transmitted infections in both partners; (e) voluntary medical male circumcision for HIV-negative men to reduce their risk of HIV acquisition; and (f) semen insemination or other assisted reproductive interventions.

## Discussion

This special supplement highlights the needs of couples affected by HIV who desire children and explores opportunities for the integration of fertility care and HIV treatment and prevention into existing healthcare services. A compelling argument to incorporate fertility services within the broader framework for integration of family planning (FP) and HIV services is presented by Mason et al. [7]. Addressing sexual and reproductive health options, Safier et al. [8] present decades of clinical research about assisted reproductive medicine interventions that achieve pregnancy with zero transmission [20–23] and the importance of assessing fertility in order to support partners to become pregnant or to make an early diagnosis of a fertility problem or infertility. To achieve these outcomes, there is a critical need to directly link HIV services with fertility and infertility care services. This level of integration will ensure that clients desiring a child will be able to limit the time during which they are at risk of exposure to HIV as well as reduce the time to achieve their fertility desires and become pregnant. Once pregnancy is confirmed in a woman living with HIV, she requires immediate referral for HIV treatment designed to eliminate perinatal transmission of HIV and to receive appropriate antenatal and perinatal care [6,9]. Clinical and research data for assisted fertility interventions resulting in pregnancy should provide child health outcomes, which includes data on transmission of infection or disease [10]. Seidman et al. [11] describe the rationale and current research evidence for the safety of tenofovir-based PrEP for HIV prevention during pregnancy and lactation and also identify the need for more implementation research in diverse clinical settings [12].

To foster communication between fertility and HIV specialists, accurate consensus-driven terminology should be used to define clients, services, interventions or outcome and used consistently during counselling. An example is the commonly used acronym, which can refer to antiretroviral treatment for HIV, and also assisted reproductive technologies. Terms, and positive language in particular, also matter to clients, as presented by women living with HIV in the article by Orza et al. [13,14]; as an example, some women who are in a relationship within which only one partner has been diagnosed as HIV positive prefer to be defined within a “serodifferent” partnership rather than a partnership described as “serodiscordant”.

Saleem et al. [15] describe a social ecological framework to characterize the interrelated opportunities at multiple levels that need to be implemented in order to ensure a rights-based approach to the integration of sexual reproductive health and rights and HIV services for people in serodiscordant relationships who desire pregnancy. The commentary by Orza et al. [13], from the perspective of women living with HIV, reports that few women feel empowered to have “self-determination regarding their sexual and reproductive health and rights”. Similarly, when presenting the perspectives of men living with HIV who desire children, Fransen-dos Santo and Guarinieri [16] state, “Public health reasoning or simple stigma (experienced and internal) had greater weight than principles of human rights and gender equity, making it especially challenging for men living with HIV to even consider accessing sexual and reproductive health services. And this is a challenge we have as yet not fully addressed.” These and other authors in this supplement describe the stigma, discrimination and violence experienced within the healthcare setting by people living with or affected by HIV including when diagnosed with fertility problems. In this context, getting appropriate information, services and care makes it even more daunting to realize their right to a desired and safe pregnancy.

Several research papers in this supplement present services and interventions in settings with high HIV prevalence, specifically in South Africa [17,18] and in Kenya [19]. Davis et al. [17], and Schwartz et al. [18], address the barriers and enablers for integration of safer conception at different levels in healthcare systems – an HIV clinic and a primary care centre, respectively. While the lack of financial support to provide any assisted reproductive medicine for many people is an issue in low-resource settings, it may be particularly acute for people living with or affected by HIV. Ngure et al. [19] note assisted reproductive medicine options based on interviews with healthcare providers and clients in HIV-serodiscordant relationships in Kenya, in contrast to antiretroviral medications which are typically provided free of cost and thus were accessible to clients desiring pregnancy. Mason et al. [7] strongly encourage that currently supported antiretroviral HIV programmes include safer conception needs, but sustainability and increased access to both antiretroviral medications and assisted reproductive medicine interventions need to be addressed.

In a global survey on sexual and reproductive health priorities of women living with HIV, the expressed desire of respondents was that they be supported to realize their fertility desires and be provided advice on safe conception, pregnancy and childbirth, coupled with equitable access to either antiretroviral or fertility treatment to attempt to have a biologically related child or access to legal adoption services [14]. They articulated a need for access to accurate, up-to-date information on options and risks of HIV transmission to partners when attempting to become pregnant as well as on the risk of perinatal HIV transmission to their children. Survey respondents emphasized the importance of support from trusted doctors and other healthcare providers in realizing their fertility desires and of peer support from other women living with HIV who have had children.

## Conclusions

The articles in this supplement reveal that rights-based, comprehensive and integrated strategies better support safe pregnancy outcomes for people in HIV-serodiscordant relationships than more traditionally “siloed” reproductive health and HIV interventions. More grassroots advocacy is required to engage and empower people living with HIV to assist in the development of zero-HIV transmission fertility care policies and programmes. People living with HIV, who are with or without fertility problems, should be provided an opportunity to decide and indicate a preference for the intervention they desire to build a family, without stigma or discrimination that best ensures the recent targets of Start Free, Stay Free and AIDS Free framework [24].

Additional research is needed on cost-effectiveness and implementation of interventions in different settings. This includes generating evidence that will evaluate and further refine integration strategies into health systems that incorporate antiretroviral and fertility interventions from settings other than those with high HIV incidence. This will help to develop evidence-informed services that are comprehensive, acceptable and affordable and provide equitable access of interventions that can meet the fertility intentions and desires of people living with or affected by HIV.
